# Impact of educational interventions on the prevention of influenza: A systematic review

**DOI:** 10.3389/fpubh.2022.978456

**Published:** 2022-09-20

**Authors:** Mohammad Javad Nasiri, Bardia Danaei, Niloofar Deravi, Alireza Salimi Chirani, Amir Hashem Shahidi Bonjar, Zohreh Khoshgoftar, Forouzan Karimi

**Affiliations:** ^1^Department of Microbiology, School of Medicine, Shahid Beheshti University of Medical Sciences, Tehran, Iran; ^2^Department of Biomedical Sciences, University of Windsor, Windsor, ON, Canada; ^3^Clinician Scientist of Dental Materials and Restorative Dentistry, School of Dentistry, Shahid Beheshti University of Medical Sciences, Tehran, Iran; ^4^Virtual School of Medical Education and Management, Shahid Beheshti University of Medical Sciences, Tehran, Iran; ^5^Department of Immunology, School of Medicine, Shahid Beheshti University of Medical Sciences, Tehran, Iran

**Keywords:** influenza, education, prevention, vaccination, systematic (literature) review

## Abstract

**Introduction:**

Seasonal influenza, a contagious viral disease affecting the upper respiratory tract, circulates annually, causing considerable morbidity and mortality. The present study investigates the effectiveness of educational interventions to prevent influenza.

**Methods:**

We searched PubMed/Medline, Embase, and Cochrane Controlled Register of Trials (CENTRAL) for relevant clinical studies up to March 1 2022. The following terms were used: “influenza,” “flu,” “respiratory infection,” “prevent,” “intervention,” and “education.”

**Results:**

Out of 255 studies, 21 articles satisfied the inclusion criteria and were included in our study: 13 parallel randomized controlled trials (RCT) studies, two cross-over RCT studies, two cohort studies, and four quasi-experimental studies. A total of approximately 12,500 adults (18 years old or above) and 11,000 children were evaluated. Educational sessions and reminders were the most common interventions. The measured outcomes were vaccination rates, the incidence of respiratory tract infection (RTI), and preventive behaviors among participants. Eighteen out of 21 articles showed a significant association between educational interventions and the outcomes.

**Conclusions:**

The included studies in the current systematic review reported the efficacy of health promotion educational interventions in improving knowledge about influenza, influenza prevention behaviors, vaccination rates, and decreased RTI incidence regardless of the type of intervention and the age of cases.

## Introduction

Health literacy is a flourishing research and practice field concerned with people's capacities to meet the complex demands of health across the course of life in our modern society, which also considers how people find, comprehend, evaluate, use, and communicate health information (1). Health literacy bears great significance in improving the prevention and control of infectious diseases. Therefore, improving the health literacy of society on infectious diseases could serve as an essential factor in controlling outbreaks and epidemics of infectious diseases worldwide (2). Health education can improve people's knowledge of contagious diseases and consequently promotes the development of appropriate preventive behaviors toward contagious disease. Health education facilitates health promotion and effectively slows down the spread of infectious diseases (2, 3).

Seasonal influenza, an infectious viral disease affecting the upper respiratory tract, circulates annually, causing considerable morbidity and mortality, mainly among adults aged ≥ 65 years and children aged < 5 years. Every year ~3 to 5 million cases of severe illness and about 290,000–650,000 respiratory deaths worldwide due to influenza infection are reported (4). The majority of influenza-related mortality (accounting for over 85% of deaths) occurs in adults older than 65 years (5); it is also associated with about 610,000–1,237,000 respiratory hospitalizations among children younger than 5 years worldwide annually (6).

Some randomized controlled trials (RCTs) have been undertaken to determine the efficacy of the educational intervention on influenza prevention (defining its impact on respiratory tract infection incidence, vaccination rate, and improvement of knowledge or preventive behaviors as the primary outcome). However, the results have been inconsistent (3, 7–10). Furthermore, there has been no systematic review investigation educational intervention on influenza prevention to the best of our knowledge. Therefore, this systematic review aimed to assess the studies reporting on the effect of education on influenza prevention.

## Methods

This review conforms to the “Preferred Reporting Items for Systematic Reviews and Meta-Analyses” (PRISMA) statement (11).

### Search strategy

The English medical literature search was carried out in PubMed/Medline, EMBASE, and the Cochrane Controlled Register of Trials (CENTRAL) up to February 1 2022.

We used the following MeSH terms: “‘Influenza, Human,' ‘Influenza B virus,' ‘Influenza A virus,' ‘education'.” Keyword searches were done with combinations of the terms: “influenza,” “flu,” “respiratory infection,” “prevent,” “early intervention,” “education,” “educate,” “school,” “school-based” and “inform” (See Appendix). Lists of references of selected articles and relevant review articles were hand-searched to identify further studies.

### Study selection

The records found through database searching were merged, and the duplicates were removed using EndNote X7 (Thomson Reuters, Toronto, ON, Canada). Two reviewers independently screened the records by title/abstract and full text to exclude those unrelated to the study topic. Clinical studies investigating the relationship between education and influenza were selected. We included RCTs, cohort studies, and case-control studies conducted on healthy individuals who had the capability to understand educational interventions.

Educational interventions can consist of informative contents such as informational e-mails, messages, reminders, virtual or in person educational sessions about hand and respiratory hygiene related to influenza and any means to inform participants about influenza.

The primary outcome assessed was the occurrence of respiratory tract infection (RTI) episodes, vaccination rates, or improvement of knowledge and preventive behaviors among participants. The exclusion criteria were: conference abstract, case report, and reviews.

Other exclusion criteria were receiving any education related to influenza, hand and respiratory hygiene by control group.

### Quality assessment

Two reviewers Bardia Danaei and Niloofar Deravi assessed the quality of the studies independently using two different assessment tools; The Newcastle-Ottawa Scale (NOS) for observational studies and the Cochrane tool for experimental studies (12, 13).

Third reviewer Mohammad Javad Nasiri was planned to decide if the two reviews couldn't able to agree on a certain point of bias assessment.

The NOS scale evaluates the risk of bias of prospective studies with three domains: (1) selection of participants, (2) comparability, and (3) outcomes. A study can be awarded a maximum of one point for each numbered item within the selection, and outcome categories and a maximum of two points can be given for comparability. Scores of 0–3, 4–6, and 7–9 were assigned for the low, moderate, and high quality of studies, respectively.

The Cochrane tool is based on; use of random sequence generation; concealment of allocation to conditions; blinding of participant and personnel; blinding of outcome assessors; completeness of outcome data and other; selective reporting and other biases. Each study was rated as low risk of bias when there was no concern regarding bias; as high risk of bias when there was concern regarding bias; or unclear risk of bias if the information was absent.

### Data extraction

Two reviewers designed a data extraction form and extracted data from all eligible studies, resolving differences by consensus. The following data were extracted: first author, country of origin, type of study, inclusion period, the definition of case and control, age group and sex of cases and controls, and the total number of controls and cases, type of intervention, and outcomes.

Extracted outcomes were categorized into three groups: (1) RTI incidence reported by studies and diagnosed with clinical features or laboratory tests such as RT-PCR, (2) Vaccination rate and uptake among participants, (3) Improvement of knowledge toward influenza or influenza prevention behaviors which were reported as compliance based on charting documentation, questionnaires and surveys.

## Results

**Figure 1:**
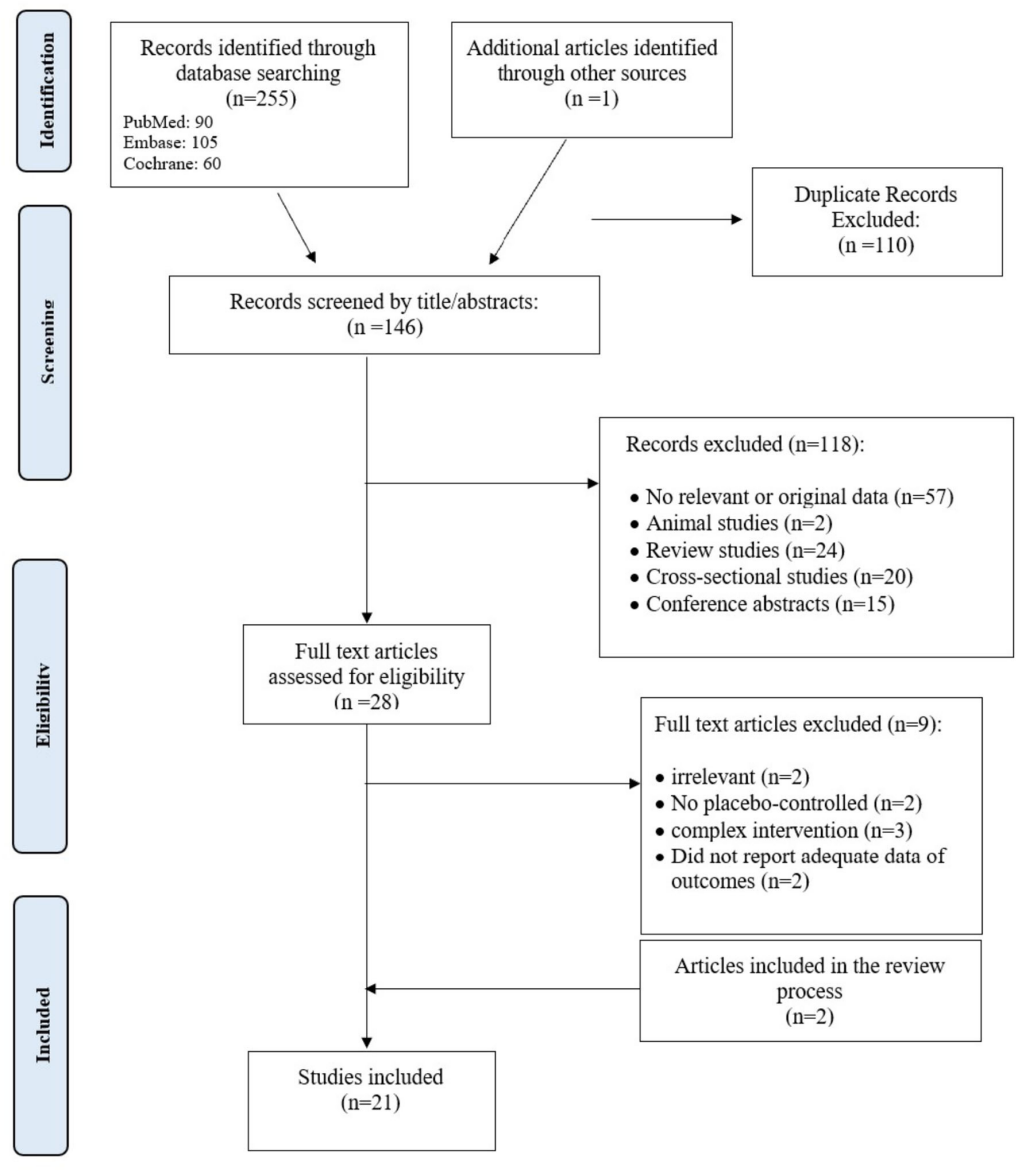
Flow chart of the number of studies identified and selected into the systematic review and meta-analysis.

Twenty-one articles were included via the selection process, which is shown in Figure 1. The studies were classified into 13 parallel RCT studies, two cross-over RCT studies, two case-control studies, and four quasi-experimental studies. Ten of these studies were conducted in the USA, two in Hong Kong, two in Iran, and others in England, Bangladesh, Greece, Australia, South Korea, China and Colombia. The population of these articles consists of approximately 12,500 adults (18 years old or above that) and 11,000 children. The outcome assessment was different between articles. Although the educational interventions were highly variable and often multimodal, all but three studies (8, 14, 15) addressed and emphasized that educational interventions are successful in influenza prevention in terms of respiratory tract infection incidence (16–22), vaccination rate (7, 9, 23–25), and improvement of knowledge or preventive behaviors (9, 26–30) (Tables 1–3).

**Table 1:** The association of educational interventions on RTI incidence.

**Author**	**Year**	**Country**	**Type of study**	**Participants' age category**	**Educational intervention type**	**Association of educational intervention on RTI incidence reduction**
Little et al. (16)	2015	England	Parallel RCT	Adult	Reminders and informative content	Yes
Biswas et al. (18)	2015	Bangladesh	Parallel RCT	Children	Educational sessions about hand and respiratory hygiene	Yes
Cowling et al. (14)	2009	Hong Kong	Parallel RCT	Both	Educational sessions about hand and respiratory hygiene	No^*^
Aelami et al. (19)	2015	Iran	Parallel RCT	Adult	Educational sessions about hand and respiratory hygiene	Yes
Aiello et al. (20)	2010	USA	Parallel RCT	Adult	Educational sessions about hand and respiratory hygiene	Yes
Stedman-Smith et al. (21)	2015	USA	Parallel RCT	Adult	Educational sessions about hand and respiratory hygiene	Yes
White et al. (22)	2014	USA	Parallel RCT	Adult	Educational sessions about hand and respiratory hygiene	Yes
Correa et al. (31)	2008	Colombia	Parallel RCT	Children	Educational sessions about hand hygiene	Yes
Stebbins et al. (15)	2008	USA	Parallel RCT	Children	Educational sessions about hand and respiratory hygiene	No^#^
Or et al. (17)	2019	Hong Kong	Quasi-experimental	Both	Educational sessions and informative content	Yes

* In short period of time (< =36days) it has been proven to be effective (P = 0.04) but in longer period it does not have a significant association (p = 0.22).

# They found no significant effect of the intervention on the outcome of all laboratory confirmed influenza (influenza A and B) cases but laboratory-confirmed influenza A infection rate in intervention schools was significantly lower.

**Table 2:** The association of educational interventions on vaccination rate.

**Author**	**Year**	**Country**	**Type of study**	**Participants' age category**	**Educational intervention type**	**Association of educational intervention on vaccination rate**
Kempe et al. (24)	2015	England	parallel RCT	Adult	One reminder and informative content	Yes
Kempe et al. (24)	2015	England	parallel RCT	Adult	Two reminders and informative content	Yes
Kempe et al. (24)	2015	England	parallel RCT	Adult	Three reminders and informative content	Yes
Szilagyi et al. (25)	2019	USA	Parallel RCT	Both	One reminder and informative content	Yes
Szilagyi et al. (25)	2019	USA	Parallel RCT	Both	Two reminders and informative content	Yes
Szilagyi et al. (25)	2019	USA	Parallel RCT	Both	Three reminders and informative content	Yes
Kopsidas et al. (7)	2020	Greece	Parallel RCT	Adult	Educational sessions and informative content	Yes
Schensul et al. (23)	2009	USA	Parallel RCT	Adult	Educational sessions with ongoing financial and vaccination support	Yes
Ferguson et al. (9)	2010	Australia	Cross over RCT	Both	Educational sessions and informative content	Yes
Bourgeois et al. (8)	2008	USA	Parallel RCT	Adult	Informative content	No

**Table 3:** The association of educational interventions on the improvement of knowledge or preventive behaviors.

**Author**	**Year**	**Country**	**Type of study**	**Participants' age category**	**Educational intervention type**	**Association of educational intervention on knowledge and preventive behaviors**
May et al. (26)	2010	USA	Case-control	Adult	Educational sessions and informative content	Yes
Ferguson et al. (9)	2010	Australia	Cross over RCT	Both	Educational sessions and informative content	Yes
Bourgeois et al. (8)	2008	USA	Parallel RCT	Adult	Informative content	No
Koep et al. (29)	2013	USA	Case-control	Children	Educational sessions and informative content	Yes
Kim et al. (27)	2020	South Korea	Quasi-experimental	Adult	Educational sessions and informative content	Yes
Sadeghi et al. (28)	2017	Iran	Quasi-experimental	Adult	Educational sessions and informative content	Yes
Wangvv et al. (30)	2016	China	Quasi-experimental	Children	Educational sessions and informative content	Yes

The setting of these studies varied from the household setting (*n* = 4) (14, 16, 23, 27), over the hospital and healthcare facilities (*n* = 7) (7, 9, 10, 24–26, 28), schools (*n* = 6) (15, 17, 18, 29–31), and University setting (*n* = 2) (20, 22), to the corporation worksites (*n* = 2) (8, 21) and one study was performed on Hajj pilgrims (19).

All 21 studies implemented at least one intervention of a promotional program with educational sessions or informative content and reminders. The educational sessions were characterized by education of the target group through either verbally communicated hand and respiratory hygiene lessons (e.g., instructions by telephone, on the internet, or face-to-face), with training or instructions on how and how frequent to practice hand hygiene and to use face masks or in combination with written or visual media (e.g., information leaflets, posters, video/live demonstration) (7, 9, 15, 17–20, 31). In addition to that, some studies consisted of the provision of hand hygiene materials, either soap (14, 19), alcohol-based hand sanitizers (14, 15, 18–20, 31), face masks (14, 19, 20), provided by the researchers to every participating individual (14, 19, 20) or to be shared within their cluster or with others in case of provision at a common place (e.g., common courtyards or school toilets) (15, 18, 31).

### Quality of included studies

Based on the Newcastle-Ottawa Scale, which was used to evaluate the quality of the observational studies, the mean (standard deviation [SD]) NOS score was 8.0, which is suggestive for a high methodological quality and a low risk of bias of the included studies. More detailed information about the quality assessment of the observational studies can be seen in Table 4.

**Table 4:** Quality assessment of the observational studies included in the meta-analysis (The NOS tool).

**Author**	**Selection**			**Comparability**			**Outcome**		
	**Represent ativeness of Exposed cohort**	**Selection of non-exposed cohort**	**Ascert ainment of exposure**	**Demonst ration that outcome of interest was not present at start of study**	**Adjust for the most important risk factors**	**Adjust for other risk factors**	**Assessment of outcome**	**Follow-up length**	**Loss to follow-up rate**	**Total quality score**
Koep et al. (29)	1	1	1	1	1	0	1	1	1	8
May et al. (26)	1	1	1	1	1	0	1	1	1	8

Risk of bias assessment of the experimental studies according to the Cochrane tool is presented in Table 5. Only four studies (9, 17, 28, 30) have a high risk of bias in the cases of allocation concealment, blinding of participants, and blinding of outcome (Table 5).

**Table 5:** Quality assessment of the experimental studies included in the meta-analysis (the Cochrane tool).

**Author**	**Random sequence generation**	**Allocation concealment**	**Blinding of participants and personnel**	**Blinding of outcome assessment**	**Incomplete outcome data**	**Selective reporting**	**Other bias**
Little et al. (16)	Low risk	Low risk	Low risk	Low risk	Low risk	Low risk	Low risk
Biswas et al. (18)	Low risk	Low risk	High risk	Low risk	Low risk	Low risk	Low risk
Cowling et al. (14)	Low risk	Low risk	High risk	Low risk	Low risk	Low risk	Low risk
Aelami et al. (19)	Low risk	Low risk	High risk	Low risk	Low risk	Low risk	Low risk
Aiello et al. (20)	Low risk	Low risk	High risk	Low risk	Low risk	Low risk	Low risk
Stedman-Smith et al. (21)	Low risk	Low risk	Low risk	Low risk	Low risk	Low risk	Low risk
White et al. (22)	Low risk	Low risk	Low risk	Low risk	Low risk	Low risk	Low risk
Or et al. (17)	High risk	Low risk	High risk	Low risk	Low risk	High risk	Low risk
Kempe et al. (24)	Low risk	Low risk	Low risk	Low risk	Low risk	Low risk	Low risk
Szilagyi et al. (25)	Low risk	Low risk	Low risk	Low risk	Low risk	Low risk	Low risk
Kopsidas et al. (7)	Low risk	Low risk	Low risk	Low risk	Low risk	Low risk	Low risk
Schensul et al. (23)	Low risk	Low risk	Low risk	Low risk	Low risk	Low risk	Low risk
Ferguson et al. (9)	High risk	High risk	High risk	Low risk	Low risk	Low risk	Low risk
Bourgeois et al. (8)	Low risk	Low risk	Low risk	Low risk	Low risk	Low risk	Low risk
Kim et al. (27)	Low risk	Low risk	Low risk	Low risk	Low risk	Low risk	Low risk
Sadegh et al. (28)	High risk	Low risk	Low risk	Low risk	Low risk	Low risk	Low risk
Wang et al. (30)	High risk	Low risk	Low risk	Low risk	Low risk	Low risk	Low risk
Correa et al. (31)	Low risk	Low risk	High risk	Low risk	Low risk	Low risk	Low risk
Stebbins et al. (15)	Low risk	Low risk	High risk	Low risk	Low risk	Low risk	Low risk

### Association of educational intervention on RTI incidence

In a study conducted by Little et al., access to a weekly automated web-based intervention for 4 weeks resulted in fewer respiratory infections in the intervention group in 4 months follow up (*P* < 0.0001) (16). In another study, hand and respiratory education with hand sanitizers supplementation in a school for 4 weeks resulted in lower RTI incidence in the intervention group (*P* < 0.01) (18). In the study conducted by Cowling et al. (14) an intervention group received hand and respiratory hygiene education, and another intervention group received education plus face masks. The control group received only lifestyle education. In 36 weeks, follow-up, there were significant differences between RT-PCR confirmed influenza cases (*P* = 0.04), but after that, the difference became non-significant (*P* = 0.283) (14). In another study conducted on Iranian hajj pilgrims in 2012, educational intervention with personal hygiene packages including alcohol-based hand rubs, surgical face masks, soap, and paper handkerchiefs resulted in significantly less Influenza-like illness (ILI) in the intervention group (*P* < 0.001). In this study, ILI was defined as at least two of the following during their stay: fever, cough, and sore throat (19). Aiello et al. (20) study included young adults from university residence halls during the 2006–2007 influenza seasons. The intervention group received respiratory hygiene education with face masks and hand hygiene equipment, resulting in significantly reduced ILI compared to the control group (*P* < 0.05) (20). Stedman-Smith et al. prepared a 4-min online training video and three brief monthly surveys as the intervention, which caused a statistically significant relative reduction of 31% in acute respiratory infections and influenza-like illnesses (*P* = 0.037) (21). In the White et al. study, participants in the experimental halls were exposed to a health campaign designed to increase awareness of the importance of hand hygiene in avoiding the flu. The intervention and control groups were both supplied with hand sanitizers. After 8 weeks of follow-up, the experimental group reported 26% fewer ILI than the control group (*P* < 0.0001) (22). Correa et al. (31) conducted a parallel RCT study on school children in which the researchers provided alcohol-based hand rubs and training on proper use for intervention group with 30-minute monthly sessions for 9 months. They reported significant reductions in risk for acute respiratory infections in the second and third trimester (*P* < 0.05, *P* < 0.001, respectively) (31). In another parallel RCT study conducted by Stebbins et al. (15) on students of 10 elementary schools, the researchers implemented an educational program called “WHACK the Flu” in intervention schools in addition to providing alcohol-based hand sanitizers. The researchers used RT-PCR to confirm influenza cases. They found no significant effect of the intervention on the outcome of all laboratory confirmed influenza (influenza A and B) cases but they reported significant fewer laboratory-confirmed influenza A infection in intervention schools. In Or et al. (17) quasi-experimental study training program for kindergarteners and their parents led to fewer flu-like signs and symptoms after the intervention (*P* = 0.005).

### Association of educational intervention on vaccination rate

In the study conducted by Kempe et al. (24) one to three reminders were sent to the parents of children in Colorado and New York primary care practices. The main outcome was documentation of ≥1 influenza vaccine within 6 months. There was no significant difference in Colorado in the one reminder and three reminders intervention group, but there was a significant increase in vaccination rates in all other intervention groups. In the Szilagyi study, patients due for an influenza vaccine in the intervention group were sent a letter reminding them about the importance of influenza vaccination, its safety, and morbidity associated with influenza. There was a statistically significant increase in vaccination rates among the intervention group (*P* = 0.008) (25). In another study, healthcare Workers participated in surveys and were educated about vaccination, and the result of intervention was evaluated by comparing the vaccination rates of the year of the intervention with the rates of the 2 years before and the year after. The intervention led to an increase in seasonal influenza vaccine uptake by the healthcare workers (*P* < 0.001) (7). In another study conducted by Schensul et al., there was a significant increase in vaccination rates among participants in a twice-a-week informative meeting held for 2 months (*P* = 0.01) (23). In the Ferguson et al. study, a 5-min education session was developed to inform patients/caretakers about the risk of respiratory viral infection, preventive measures, and efficacy. The primary outcome was awareness of and attitudes toward preventive strategies, and the vaccination rate significantly increased in the intervention group (*P* < 0.0001) (9). In the study conducted by Bourgeois et al., participants were recruited from eight Hewlett Packard Corporation work sites in the northeastern United States in the fall of 2005. They evaluated an electronic system to modify knowledge, beliefs, and behavior around influenza. They sent weekly messages for 4 weeks to the intervention group, but there was no significant difference in the immunization rate between the intervention and control groups (*P* = 0.5) (8).

### The association of educational interventions on the improvement of knowledge or preventive behaviors

In the study conducted by May et al. the investigators sent an informational e-mail to healthcare providers *via* an electronic medical record system, explaining the importance and proper use of infection control precautions for patients with clinically suspected or confirmed influenza. The email was repeated every month for 7 months for the intervention group. After this intervention, the overall compliance with transmission precautions increased (*P* = 0.015) (26). As mentioned above, Ferguson et al. provided a 5-min education session to participants in the intervention group to increase their awareness and attitude toward preventive measures which evaluated by self-administered questionnaires (P < 0.0001) (9). Bourgeois et al. sent weekly messages to participants in the intervention group to modify knowledge, beliefs, and behavior around influenza for 4 weeks which could not improve their knowledge and preventive behaviors significantly compared to the control group (*P* = 0.5). Surveys containing questions on influenza knowledge, beliefs, and behavior was used to collect data. (8). In the study conducted by Koep et al. (29) teachers and students were recruited to participate in 4 to 6 weeks of the internship, including education about vaccine design and effect, hand hygiene and cough etiquette, germ growth, and immune system functioning. Talking drawings (TD's) strategy were used to assess pre/ post intervention changes in students understanding and emerging student language. The results showed improvement in influenza prevention understanding (*P* < 0.0006) (29). In another quasi-experimental study conducted by Kim et al. on rural elderly individuals, a health education program aimed at preventing respiratory infections with 4 weekly sessions and a reinforcement session one to 6 months after the initial training led to a significant increase in respiratory infection prevention practices (*P* < 0.001). In this study the respiratory infection prevention knowledge scale developed by Kwon and Yu were used for measurement of knowledge (27, 32). In a quasi-experimental study conducted by Sadeghi et al. (28) in Iran on pregnant women, informative content including the definition of flu, its symptoms, transmission, prevention, diagnosis, and treatment was educated within 2 weeks to the intervention group, which caused significant improvement in knowledge and preventive behaviors (*P* = 0.001). Health Belief Model Questionnaire was used in this study (28). In another quasi-experimental study, Wang et al. designed an education program for children, including playing promotion cartoons, developing lectures, giving out handbook copies, and making hand copy and blackboard newspapers, which held monthly for one year and led to significant improvement of behavior toward respiratory infectious disease (RID) in the intervention group (*P* < 0.05). Authors used a self-designed questionnaire including items concerning knowledge on RID and RID prevention behavior for in this study (30).

## Discussion

The present study found that educational interventions show efficacy for influenza prevention by impacting respiratory tract infection incidence, vaccination rate, and improvement of knowledge or preventive behaviors. Several explanations were reported, which are summarized in the following.

A study in Greece aimed to increase healthcare workers' uptake of seasonal influenza vaccination in a tertiary-care pediatric hospital with a low-cost, tailor-made, multifaceted strategy. This strategy increased the vaccination rate by overcoming barriers such as concerns about the safety and effectiveness of the vaccine, the belief that one does not belong to a high-risk group, logistical concerns, and concerns about side effects of the vaccine (7). Another study in the United States aimed to increase residents' ability to make informed decisions about vaccination and build internal and external infrastructure to support sustainability over time. Evaluation results showed that the educational intervention achieved most of its desired goals at all levels. It achieved significantly increased pro-vaccination knowledge, beliefs, and norms with increased correct social thoughts toward vaccination. It reduced vaccination fears among peer implementers and participating residents and finally improved the vaccination rate in the intervention building (23).

A South Korean study reported highly positive outcomes of an educational program for respiratory infection prevention among rural elderly residents (27). The reasons for the program's high efficacy were that it was developed based on the processes of observational learning (i.e., attention, memory, retention, and motivation). Key concepts from this theory, such as imitation, cognition, reinforcement, and self-efficacy, were also applied to the development of the program. One of the four observational learning processes, attention, was incorporated in the program in such a way as to enhance trainees' interests and motivation: the trainees were asked to think about why the topic of each training session was necessary. They were also asked to reflect on what they had learned by singing a song or taking a pledge of practice. The memory process, which involves the cognitive storage of information learned from observation, was promoted in the program through video materials used to stimulate both vision and hearing among the trainees. To enhance their concentration, various instruments were used, such as a dental model, a view box, masks, and a walking mat. These instruments helped to demonstrate actions like correct hand washing, correct walking, etc. Next, the retention process, which involves determining the consistency between what one learns and how one subsequently behaves through internal feedback, was invoked among trainees through self-observation and self-correction, i.e., the trainees were guided to adapt theirs to the model's behavior. As the final step, the motivation process was stimulated by encouraging trainees to engage in program practices. The trainees sang a song that included keywords and key concepts from each training session. In addition, the trainees were also asked to take a pledge of practice to review what they had learned (27).

Rewards were used to increase participation among the trainees across all sessions. At the beginning of each session, rewards were given to the trainees who shared what they had learned from the previous session with their peers. Some trainees were also rewarded with items for respiratory infection prevention such as a handkerchief, hand sanitizer, and toothbrush/toothpaste for completing tasks, memorizing the songs and pledge of practice, or correctly answering questions on the quiz. In the final session, certificates and prizes were awarded to the trainees for participating in the program. For the program, instructors were selected based on whether their ages approximated the trainees' age to enhance rapport and familiarity (27). Such an approach has previously been applied to increase compliance with respiratory infection prevention practices (33).

A Chinese study reported the efficacy of health education on the knowledge and behavior of students toward RID. According to this study, in primary, middle, and high schools in China, students are busy with various exams and lack awareness and enthusiasm for learning about RID. At the same time, Chinese schools often neglect to teach infectious disease-related courses, leading to a lack of health education toward RID among school students and hindering the improvement of the accuracy rate and scores of RID of these students. Such a phenomenon may be one of the leading causes of the outbreak and epidemic of RID in primary, middle, and high school campuses (30). Therefore, educational interventions could lead to positive outcomes in this group. An Australian study aiming to measure and increase patient/household awareness of RV infection and preventive measures suggested that patients preparing for hematopoietic stem cell transplant (SCT), and their families and friends, understood that RVs pose a risk to patients after transplantation. Still, they underestimated its severity and lacked knowledge on effective prevention methods. A brief information session nested in a broader educational forum increased knowledge and acceptability of prevention methods, particularly family influenza vaccination, with an associated increase in household vaccination evident at the time of hematopoietic SCT admission (9).

Although in another study in the United States, the intervention did not have a statistically significant effect on the knowledge elements assessed (probably due to small sample size); however, it did have a considerable effect on certain beliefs surrounding influenza. At the end of the study, participants in the intervention group were more likely to believe that the influenza vaccine was effective, that there were actions they could take to prevent the flu, and that the influenza vaccine was unlikely to cause a severe reaction (8).

To conclude, according to the results of included studies, educational interventions could be effective in preventing Influenza. We recommend using validated educational tools clearly described, widely generalizable, and easily reproducible. We also encourage cluster randomized trials to investigate the efficiency of both organizational and educational interventions and incorporate a cost assessment into the study design. In the case of non-RCTs, addressing the threats to internal validity is required (e.g., by using a parallel control group and a time-series method with multiple observations conducted both before and after interventions). Statistical analyses are encouraged to include assessments of secular trends rather than comparisons of mean values before and after educational interventions. The sample in most studies was mixed gender cohorts. Hence, to ascertain whether between-gender differences exist, future studies must include sub-analysis by gender. Notably, the lack of a detailed description of the educational strategies content, including an assessment of validation of the educational interventions, needs to be addressed. Baseline characteristics such as the learner's academic level or job may also affect the ideal type of intervention for the participants. Future studies should also address whether reminders are needed and at what intervals they should be repeated.

There are some limitations in the current systematic review. Some studies used education methods that were not easy to use by all the population. For example, older adults were less likely to use modern educational methods. Other limitations of the studies were the limited number of participants and institutions and the duration of studies. The studies also had different settings and methodologies. Likewise, due to the limited number of studies, potential factors that lead to overall heterogeneity were not examined.

In conclusion, the findings of our systematic review suggest that health promotion educational interventions, i.e., regardless of the type of intervention and the age of cases, can improve knowledge and preventive behaviors vaccination rates and decrease RTI incidence.

## Data availability statement

The original contributions presented in the study are included in the article/Supplementary material, further inquiries can be directed to the corresponding author/s.

## Author contributions

All authors listed have made a substantial, direct, and intellectual contribution to the work and approved it for publication.

## Funding

This study was supported by Shahid Beheshti University of Medical Sciences, Tehran, Iran.

## Conflict of interest

The authors declare that the research was conducted in the absence of any commercial or financial relationships that could be construed as a potential conflict of interest.

## Publisher's note

All claims expressed in this article are solely those of the authors and do not necessarily represent those of their affiliated organizations, or those of the publisher, the editors and the reviewers. Any product that may be evaluated in this article, or claim that may be made by its manufacturer, is not guaranteed or endorsed by the publisher.
