# Supplementary material for: Impact of educational interventions on the prevention of influenza: A systematic review
Source: Front Public Health. 2022 Sep 20;10:978456. doi: 10.3389/fpubh.2022.978456 (PMC9530567; doi:10.3389/fpubh.2022.978456)
Supplement: Supplementary file 1 [file Table_1.docx]

Appendix**. Search strategy**

Table 1. Search strategy for PubMed/Medline

| **database** | **Search terms** | **Results** |
| --- | --- | --- |
| PubMed/Medline | (influenza[tiab] OR flu[tiab] OR "respiratory infection"[tiab] OR "Influenza, Human"[Mesh] OR "Influenza B virus"[Mesh] OR "Influenza A virus"[Mesh]) AND (prevent[tiab] OR "early intervention") AND (education[tiab] OR educate[tiab] OR school[tiab] OR school-based[tiab] OR inform[tiab] OR “education”[Mesh]) AND (intervention[tiab] OR RCT[tiab] OR "controlled trial"[tiab] OR randomized[tiab] OR random[tiab] OR Randomly[tiab] OR Placebo[tiab] OR Assignment[tiab] OR "clinical trial"[tiab] OR trial[tiab] OR randomized[tiab] OR "Methods"[Mesh] OR "Randomized Controlled Trial"[Publication Type] OR "Controlled Clinical Trial"[Publication Type] OR "Placebos"[Mesh] OR "Placebo Effect"[Mesh] OR "Clinical Trial"[Publication Type] OR "Clinical Trials as Topic"[Mesh]) | 90 |
