# Supplementary material for: Impact of educational interventions on the prevention of influenza: A systematic review
Source: Front Public Health. 2022 Sep 20;10:978456. doi: 10.3389/fpubh.2022.978456 (PMC9530567; doi:10.3389/fpubh.2022.978456)
Supplement: Supplementary file 2 [file Table_2.docx]

Appendix**. Search strategy**

Table 2. Search strategy for Embase

| **database** | **Search terms** | **Results** |
| --- | --- | --- |
| Embase | (influenza:ab,ti OR flu:ab,ti OR 'respiratory infection':ab,ti) AND (prevent:ab,ti OR 'early intervention':ab,ti) AND (education:ab,ti OR educate:ab,ti OR school:ab,ti OR 'school based':ab,ti OR inform:ab,ti) AND (intervention:ab,ti OR 'controlled trial':ab,ti OR random:ab,ti OR randomly:ab,ti OR placebo:ab,ti OR assignment:ab,ti OR 'clinical trial':ab,ti OR trial:ab,ti OR randomized:ab,ti) | 105 |
