# Supplementary material for: Impact of educational interventions on the prevention of influenza: A systematic review
Source: Front Public Health. 2022 Sep 20;10:978456. doi: 10.3389/fpubh.2022.978456 (PMC9530567; doi:10.3389/fpubh.2022.978456)
Supplement: Supplementary file 3 [file Table_3.docx]

Appendix**. Search strategy**

Table 3. Search strategy for CENTRAL

| **#** | **Search terms** | **Results** |
| --- | --- | --- |
| 1 | (Influenza):ti,ab,kw OR (flu):ti,ab,kw OR ("respiratory infection"):ti,ab,kw OR MeSH descriptor: [influenza, Human] explode all trees | 10550 |
| 2 | (education):ti,ab,kw OR (educate):ti,ab,kw OR (school):ti,ab,kw OR (school-based):ti,ab,kw OR (inform):ti,ab,kw OR MeSH descriptor: [Education] explode all trees | 113953 |
| 3 | (prevent):ti,ab,kw OR ("early intervention"):ti,ab,kw | 49671 |
| 4 | #1 AND #2 AND #3 | 83 |
